# Site-Specific Phosphoproteomic Profiling of CAV1 Reveals Co-Regulatory Kinase Networks in Cancer Signaling

**DOI:** 10.3390/ijms27104326

**Published:** 2026-05-12

**Authors:** Chrysilla Espy Vaz, Manasa Suresh, Leona Dcunha, Rajesh Raju, Saptami Kanekar

**Affiliations:** Centre for Integrative Omics Data Science, Yenepoya (Deemed to Be University), Mangalore 575018, India

**Keywords:** Caveolin-1, CAV1, phosphoproteomics, carcinogenesis, cell cycle regulation

## Abstract

Caveolin-1 (CAV1) is a 21 kDa Vesicular Integral-membrane Protein essential for the biogenesis of caveolae, invaginations of the plasma membrane that coordinate membrane trafficking, lipid homeostasis, and signal transduction. CAV1 functions as a scaffolding platform that integrates mechanotransduction, endocytosis, and cellular stress responses, thereby modulating vascular integrity, inflammation, metabolism, and tumorigenesis. To comprehensively understand the phosphorylation landscape of CAV1, global phosphoproteomic datasets and their corresponding experimental metadata were systematically curated and integrated from previously published human cellular studies. The phosphorylation sites with the highest detection frequency across these datasets were considered predominant phosphorylation sites. To assess their functional relevance, phosphosites in other proteins (PsOPs) co-regulated with the predominant CAV1 sites, along with their upstream kinases and high-confidence protein–protein interaction partners, were systematically analyzed. Analysis of global human cellular phosphoproteome datasets revealed that tyrosine 14 (Y14) and serine 37 (S37) of CAV1 are the most frequently detected phosphosites across diverse experimental conditions. Notably, many of the co-regulated proteins obtained were associated with carcinogenesis, apoptosis, and cell cycle regulation, including MET and ERBB2. Our analysis revealed SRC, ABL2, ERBB2, ERBB3, LYN, and TEC as potential upstream kinases of CAV1_Y14, whereas CSNK1E and GRK5 were predicted to regulate CAV1_S37. Considering the challenges associated with site-specific interrogation, we employed a global co-regulation analysis approach to characterize CAV1 phosphorylation dynamics. Our findings reveal that key CAV1 phosphosites modulate oncogenic signaling, cytoskeletal remodeling, and membrane organization, providing novel insights into CAV1-mediated cellular functions and its context-dependent role in tumor progression.

## 1. Introduction

Caveolin-1 (CAV1) is a 21 kDa protein, also known as Vesicular Integral-membrane Protein of 21 kDa (VIP21), encoded at chromosomal locus 7q31.2, which is associated with the plasma membrane. It adopts a hairpin-like topology, with both the N- and C-terminal hydrophilic domains oriented toward the cytoplasm and a central hydrophobic segment embedded within the lipid bilayer [1,2]. CAV1 is essential for caveolae formation, and its expression level correlates with the number of caveolae [3,4,5,6,7]. Caveolae, or ‘small caves’, are 50–100 nm non-clathrin, flask-shaped invaginations of the plasma membrane, functioning as specialized membrane microdomains. They were morphologically identified in 1953 by transmission electron microscopy [8]. Caveolins are a family of signature proteins of caveolae. The caveolin protein family comprises three members: CAV1, Caveolin-2 (CAV2), and the muscle-specific Caveolin-3 (CAV3) [9,10,11]. While CAV1 and CAV2 often co-localize and form hetero-oligomeric complexes in non-muscle cells, CAV3 is predominantly expressed in skeletal and cardiac muscles, where it plays critical role in muscle cell physiology and pathology. The distinct tissue distribution and specialized functions of each caveolin isoform reflect their tailored contributions to cellular architecture and signaling pathways. CAV1 displays dynamic subcellular localization patterns and is enriched in the plasma membrane, Golgi apparatus, and transport vesicles [10,12,13]. Isoform-specific targeting is partly determined by the N-terminal domain, and CAV1 can exist in cytosolic or secreted forms, depending on the cellular context [14,15]. High levels of CAV1 are found in adipocytes, endothelial cells, fibroblasts, and various epithelial cells, with CAV2 often co-expressed. In contrast, CAV3 is restricted to striated muscles [16].

Caveolin-1 exists as two primary isoforms, α and β, with the β isoform generated via an internal translational start site, resulting in the absence of the first 31 N-terminal amino acids present in the α isoform [17]. Both isoforms share a conserved domain architecture characterized by an N-terminal domain, a central hydrophobic transmembrane segment, and a C-terminal domain. Structural and mutational studies support a membrane-spanning hairpin topology in which both the N- and C-termini are oriented towards the cytoplasm, facilitating interactions with cytosolic signaling molecules and membrane lipids. Post-translational modifications critically regulate functional versatility and subcellular localization of CAV1. Palmitoylation at multiple cysteine residues enhances membrane association and stabilizes caveolar structures, whereas tyrosine phosphorylation, particularly at Tyr14, modulates involvement of CAV1 in signal transduction pathways by altering its binding affinity to signaling partners such as Src-family kinases and endothelial nitric oxide synthase (eNOS) [6,18,19,20]. These modifications influence CAV1-mediated processes, including vesicular trafficking, cholesterol transport, and spatial organization of signaling complexes.

Contemporary protein bioinformatics approaches emphasize the importance of deciphering post-translational modification landscapes to understand protein functions at the system level. Phosphorylation is a central mechanism for modulating CAV1 protein–protein interactions, subcellular localization, and signaling outputs. Therefore, large-scale phosphoproteomics and integrative bioinformatics analyses are essential for mapping the site-specific phosphorylation landscape of CAV1, ultimately advancing our understanding of how dynamic post-translational modifications orchestrate caveolae assembly, signaling specificity, and disease-associated cellular reprogramming [21,22,23,24,25].

## 2. Results

### 2.1. CAV1 Phosphoproteomics Data Overview

A comprehensive collection of 3825 publicly accessible human cell line phosphoproteomic datasets was assembled. Each dataset corresponds to a distinct biological or experimental condition reported within a published study, rather than to an entire publication; a single study may therefore contribute multiple datasets if multiple independent conditions were analyzed. From this collection, 686 qualitative profiling datasets and 163 quantitative differential datasets containing Class I phosphosites of CAV1 were identified and curated. A detailed metadata summary, including study PMIDs, number of datasets contributed per study, and corresponding phosphosite counts, is provided in Supplementary Table S3. It should be noted that, due to the limited and inconsistent availability of raw mass spectrometry files across published studies, the dataset was assembled using processed phosphoproteomic result tables obtained from published Supplementary Materials and, where available, from public repositories such as PhosphoSitePlus and ProteomeXchange. Therefore, data integration was conducted at the level of reported phosphosite identification and differential regulation as defined within each individual study without reprocessing raw mass spectrometry files. Detailed dataset information, including PMIDs and experimental condition labels, is provided in Supplementary Tables S1 and S2.

In total, 14 distinct Class I phosphosites of CAV1 were detected across the profiling datasets, with nine sites reported to be differentially regulated under at least one experimental condition. Cross-study heterogeneity arising from differences in phosphopeptide enrichment strategies (e.g., immobilized metal affinity chromatography [IMAC], titanium dioxide [TiO2], and sequential enrichment for phosphopeptides [SIMAC]), instrumentation, and data processing pipelines was addressed through the application of uniform inclusion and filtering criteria: restriction to Class I phosphosites (localization probability ≥ 75% or A-score > 13), standardization of protein identifiers to HUGO Gene Nomenclature Committee (HGNC) gene symbols, harmonization of phosphosite annotations to UniProt accession numbers, and implementation of frequency-based thresholds requiring detection across at least three independent studies.

### 2.2. Systematic Identification of Predominant CAV1 Phosphosites

**Figure 1:**
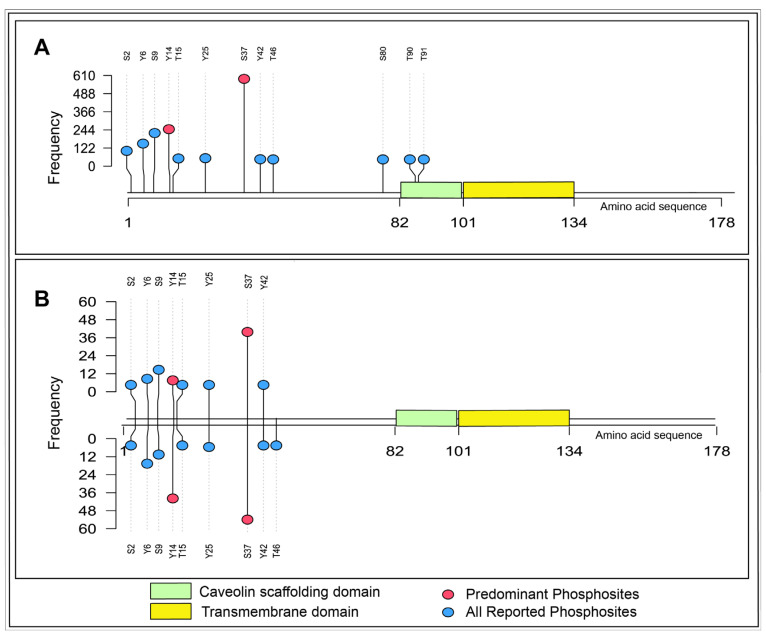
Distribution and differential regulation of CAV1 phosphosites across phosphoproteomic datasets. Lollipop plots depicting Class I phosphosites of CAV1 observed in human cellular profiling and differential phosphoproteomics datasets. The *X*-axis represents the amino acid sequence length, and the *Y*-axis represents the number of datasets in which specific Class-1 phosphosites were detected. The green region denotes the caveolin scaffolding domain, and the yellow region denotes the transmembrane domain within CAV1. Red circles indicate predominant phosphosites, while blue circles represent all reported phosphosites. (**A**) Phosphosites identified in qualitative profiling datasets. (**B**) Phosphosites identified across quantitative differential datasets. In panel (**B**), the upper lollipops represent phosphosites with increased phosphorylation frequency (upregulated relative to the study-specific reference condition), and the lower lollipops represent phosphosites with decreased phosphorylation frequency (downregulated relative to the study-specific reference condition).

To identify the predominant phosphosites of CAV1, all Class I phosphosites were ranked according to their frequency of detection—defined as the number of independent datasets in which a given phosphosite was identified—across both qualitative profiling and quantitative differential datasets. CAV1_Y14 and CAV1_S37 emerged as the most frequently detected phosphorylation sites. Specifically, CAV1_Y14 was observed in 264 profiling and 51 differential datasets, whereas CAV1_S37 was observed in 603 profiling and 97 differential datasets (Figure 1). The substantially higher detection frequency of these two sites relative to other CAV1 phosphosites, across diverse experimental systems, cell lines, and perturbation contexts, supports their designation as the predominant phosphorylation sites of CAV1 in human cellular phosphoproteomes.

To determine whether these predominant phosphosites are evolutionarily conserved across the caveolin protein family, multiple-sequence alignment was performed among CAV1, CAV2, and CAV3. The analysis demonstrated that neither Y14 nor S37 of CAV1 is conserved in CAV2 or CAV3. This finding indicates that these residues are unlikely to represent universally shared regulatory elements across the caveolin family; instead, they may contribute to isoform-specific regulatory functions unique to CAV1. From a technical standpoint, the absence of conservation at these positions also reduces the likelihood of cross-isoform confounding in phosphoproteomic detection.

### 2.3. Analysis of Co-Occurrence of Phosphorylation Within CAV1

**Figure 2:**
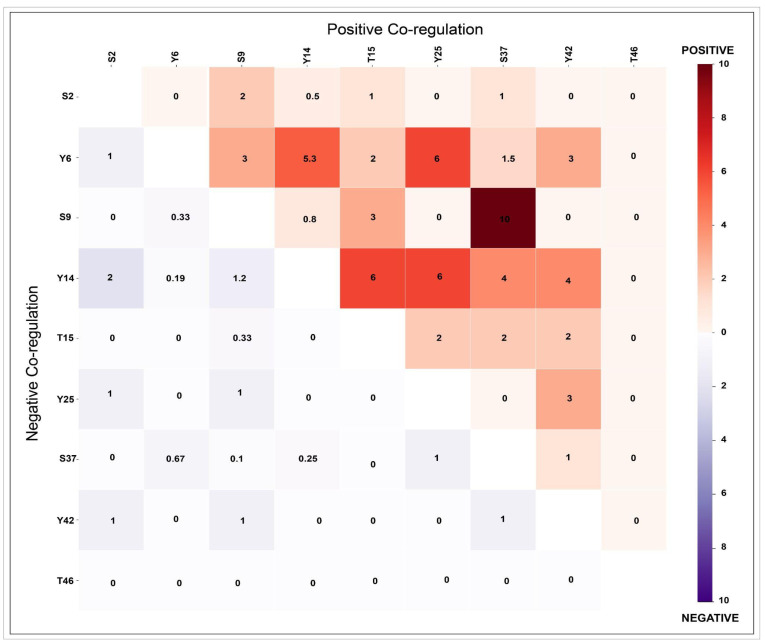
Heat map showing positive and negative co-occurrence patterns among CAV1 phosphosites. Each cell represents the degree of co-occurrence between the two sites within CAV1. Red indicates positive co-regulation, and violet indicates negative co-regulation. The numerical values represent the relative co-regulation ratios across datasets: UUDD/UDDU for positive and UDDU/UUDD for negative coregulation, reflecting the strength of the association between phosphosite pairs. Color intensity corresponds to the magnitude of the ratios, with stronger color indicating higher concordant co-regulation.

To identify the potential co-occurrence among CAV1 phosphosites, we independently quantified the frequencies of positive and negative co-regulation for each CAV1 phosphosite pair across differential datasets (Supplementary Table S4). Positive and negative co-regulation frequencies were independently quantified for each phosphosite pair, with higher ratio values indicating stronger coordinated regulation (Figure 2). The predominant phosphosites S37 and S9 demonstrated the strongest positive co-regulation, reflecting frequent simultaneous differential regulation under comparable experimental conditions (Figure 2). Moderate positive associations were observed between Y6 and Y25, whereas Y14 showed moderate positive co-regulation with Y25. These moderate associations likely reflect context-dependent or stimulus-specific regulatory relationships rather than constitutive co-regulation. Importantly, while Y14 and S37 exhibit a tendency toward concordant co-regulation in datasets where both are detected, the sets of PsOPs associated with each site are largely non-overlapping. The consistent co-differential regulation of S37 and S9 supports the hypothesis that these residues function as a coordinated phosphorylation module, potentially sharing upstream kinase regulation or being involved in common signaling pathways.

### 2.4. Phosphosites of Other Proteins (PsOPs) That Positively and Negatively Correlate with the Predominant Sites of CAV1

**Figure 3:**
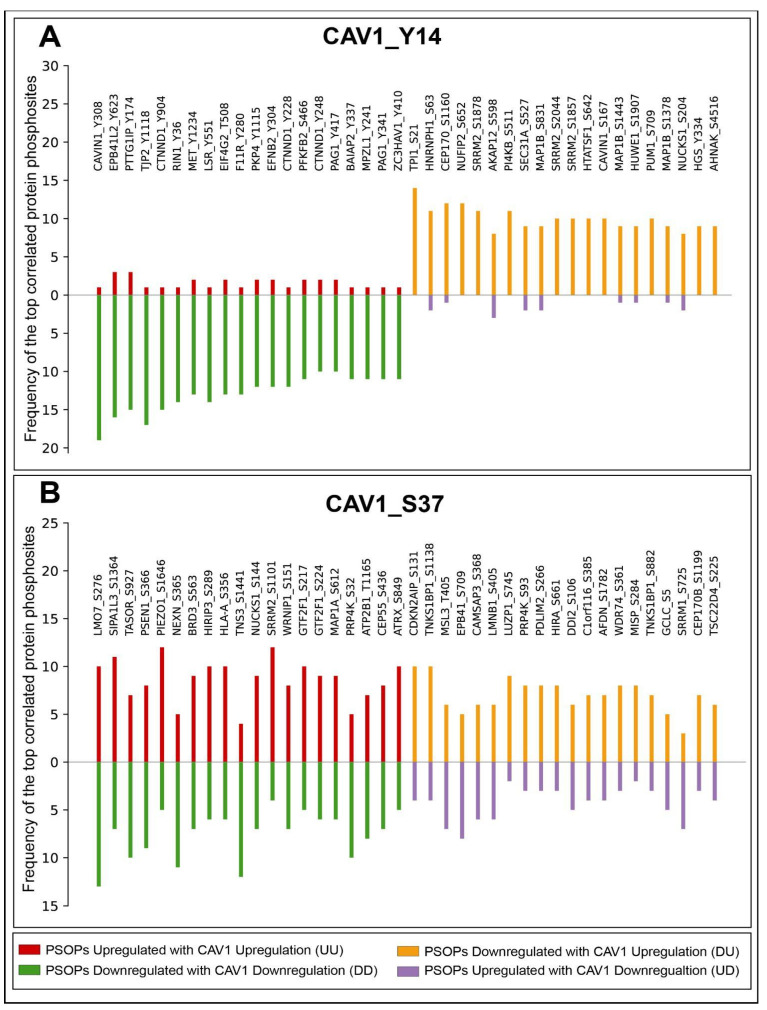
Top 20 high-confidence positively and negatively correlated PsOPs of predominant CAV1 phosphosites. (**A**) PsOPs correlated with CAV1_Y14. (**B**) PsOPs correlated with CAV1_37. Bar plots represent the frequency at which the corresponding phosphosites were identified as differentially regulated across phosphoproteomic studies. Phosphosites are categorized based on their direction of regulation relative to CAV: UU (red)—both CAV1 and the associated phosphosite are upregulated; DD (green)—both are downregulated; DU (orange)—CAV1 is upregulated, whereas the associated phosphosite is downregulated; UD (purple)—CAV1 is downregulated while the associated phosphosite is upregulated.

Co-regulation analysis can reveal biologically relevant functional relationships between proteins, irrespective of whether they physically interact or co-localize [26]. Here, we examined the PsOPs that tend to show consistent co-regulation with CAV1-predominant phosphosites to identify potential functional associations. To ensure this, we applied strict inclusion criteria to Fisher’s Exact Test (FET)-derived protein phosphosites. FET is a statistical method used to assess non-random associations between categorical variables, particularly in small sample sizes. The criteria included (i) frequency cut-off ≥10% from the total differential frequency of the predominant site for positive or negative coregulation, (ii) FET score (*p*-value) < 0.05, (iii) consistent coregulation reported in at least three independent publications (PMID confidence), and (iv) observed across a minimum of three distinct experimental conditions (code count). Using this framework, 589 positively and 242 negatively co-regulated high-confidence PsOPs were identified for CAV1_Y14, whereas 183 and 28 PsOPs showed positive and negative co-regulation with CAV1_S37, respectively (Supplementary Tables S5–S8). The top high-confidence PsOPs are shown in Figure 3. Each PsOP is categorized according to its co-regulation pattern relative to CAV1: UU (both CAV1 and the associated phosphosite upregulated), DD (both downregulated), DU (CAV1 upregulated while the associated phosphosite is downregulated), and UD (CAV1 downregulated while the associated phosphosite is upregulated). The substantially larger PsOP network associated with CAV1_Y14 compared to CAV1_S37 is consistent with the higher detection frequency and more extensive functional characterization of Y14 across phosphoproteomic datasets.

### 2.5. Biological Processes Associated with Positively and Negatively Correlated PsOPs of CAV1

**Figure 4:**
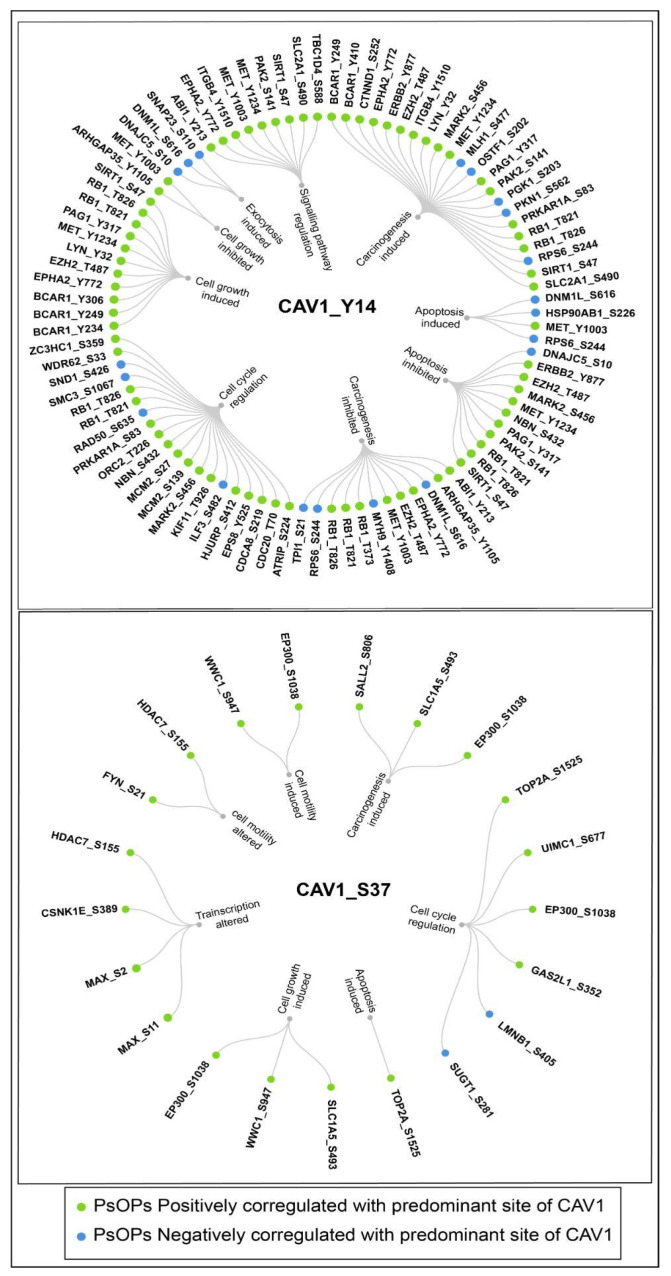
Network representation of annotated biological process associations of high-confidence coregulated PsOPs of CAV1. Peripheral nodes represent co-regulated phosphosites, with green and blue indicating positive and negative co-regulatory relationships, respectively. Phosphosites are grouped according to their reported involvement in biological processes based on curated annotations from the PhosphoSite Plus database and supported by evidence from the literature.

To characterize the functional context of the identified co-regulated phosphosite networks, PsOPs were annotated based on their reported biological process associations using curated data from the PhosphoSitePlus database, supplemented by evidence from the literature. This approach reflects descriptive, annotation-based grouping rather than formal statistical pathway enrichment analysis (e.g., overrepresentation analysis or gene set enrichment analysis). Fifty-one high-confidence PsOPs co-differentially regulated with CAV1_Y14 were linked to biological processes, including carcinogenesis, apoptosis, cell cycle regulation, signaling pathway regulation, and endocytosis. In contrast, 14 high-confidence PsOPs co-differentially regulated with CAV1_S37 were annotated to processes including cell cycle regulation, cell motility, cell growth, transcription, apoptosis, and carcinogenesis (Figure 4).

The notably broader range of biological process associations for the Y14 network relative to S37 is consistent with the higher detection frequency, broader dataset coverage, and more extensive prior functional characterization of CAV1_Y14 in phosphoproteomic sttudies. In contrast, CAV1_S37 is detected less frequently and remains comparatively under-characterized, which limits the number of high-confidence PsOPs identified and, consequently, the breadth of annotatable biological process associations. It should be noted that individual phosphosites may be associated with multiple biological processes, reflecting the inherent functional pleiotropy of signaling nodes and the overlapping nature of cellular pathways; therefore, these annotations represent potential functional connectivity rather than exclusive pathway membership. Formal functional enrichment analysis of PsOP-associated proteins revealed that the Y14-associated network was enriched in pathways related to signal transduction, cytoskeletal organization, cell adhesion, and intracellular trafficking, whereas the S37-associated network was predominantly enriched in processes governing nucleic acid metabolism, RNA regulation, and negative control of biosynthetic pathways (Supplementary Tables S11 and S12).

### 2.6. Co-Differential Regulation of Known and Predicted Upstream Kinases of CAV1

To identify upstream kinases potentially implicated in the phosphorylation of the predominant CAV1 sites, kinase–substrate relationships were curated from experimentally validated databases (PhosphoSitePlus, Phospho.ELM 9.0, RegPhos 2.0) and supplemented with in silico predictions from NetworKIN, AKID (http://akid.bio.uniroma2.it/), and iKiP-DB (HTP), as well as kinase assignments from a comprehensive synthetic peptide screening atlas (90th percentile cutoff; Johnson et al., 2023) [27]. The identified candidate kinases were subsequently cross-referenced with the PsOP co-regulation analysis to identify those exhibiting statistically significant co-differential regulation with the predominant CAV1 phosphosites across independent datasets (Supplementary Table S9). For CAV1_Y14, SRC was confirmed as an experimentally validated upstream kinase, consistent with its well-established role in CAV1 signaling. ABL2 was identified as a predicted kinase based on Johnson et al. (2023) [27], while ERBB2, ERBB3, LYN, and TEC were predicted upstream kinases exhibiting both positive and negative co-differential regulation patterns with CAV1_Y14 across datasets. For CAV1_S37, Casein Kinase 1 Epsilon (CSNK1E) and G Protein-Coupled Receptor Kinase 5 (GRK5) were identified as predicted associated kinases. It is important to note that co-differential regulation between a kinase and substrate phosphosite does not establish a direct regulatory relationship.

### 2.7. Co-Differential Regulation of the Binary Interactors of the Predominant Sites of CAV1

Phosphorylation is essential for the regulation of protein–protein interactions. To identify high-confidence interaction partners of CAV1 whose phosphosites exhibit co-differential regulation with the predominant CAV1 sites, protein–protein interaction (PPI) data were systematically extracted from six curated databases: the Human Protein Reference Database (HPRD), the Biomolecular Interaction Network Database (BIND), the Biological General Repository for Interaction Datasets (BioGRID), the Comprehensive Resource of Mammalian Protein Complexes (CORUM), RegPhos 2.0, and ConsensusPathDB (version 35) [28,29,30,31,32]. Interaction partners were retained only when supported by experimental evidence from at least one database and corroborated by cross-database support, yielding a final set of 34 high-confidence interacting proteins.

**Figure 5:**
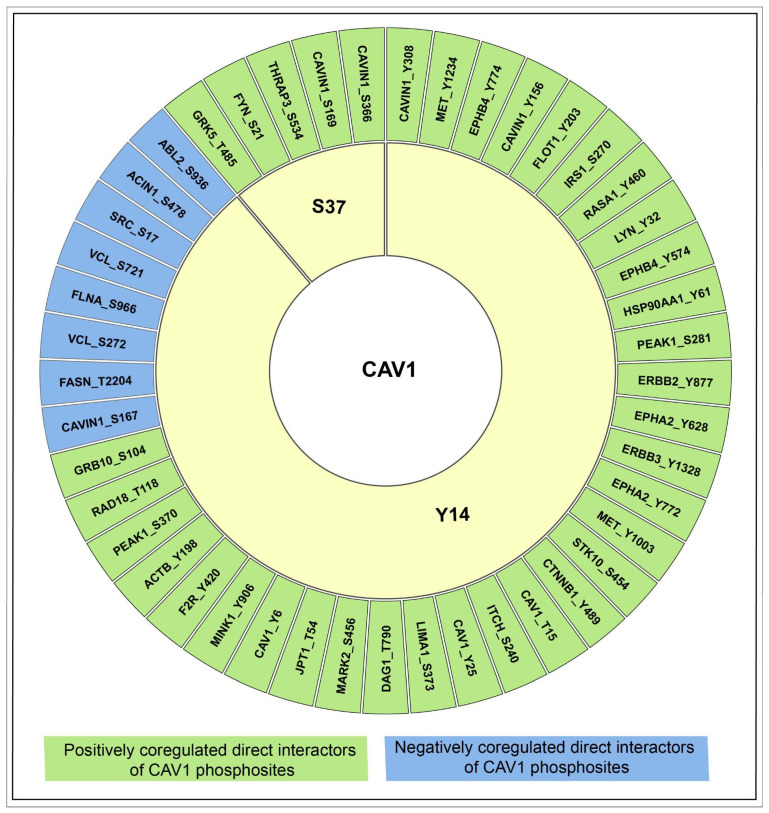
Figure depicting the predicted binary interactors of the predominant sites of CAV1. The green and blue highlights represent the positively and negatively coregulating interaction partners of CAV1, respectively.

These 34 interactors were designated as ‘high-confidence interacting proteins’ rather than ‘direct interactors’, as the supporting evidence does not uniformly reflect direct physical binding and may, in some cases, include indirect or co-complex associations. Among the interactors identified, phosphosites exhibiting significant co-differential regulation with CAV1_Y14 and/or CAV1_S37 included: CAVIN1 (Y308, Y156, S167, S169, S366), EPHA2 (Y628, Y772), ERBB2 (Y877), MET (Y1234, Y1003), SRC (S17), and Vinculin (VCL; S272, S721) (Figure 5; Supplementary Table S10). Functional enrichment analysis of the interactor-associated phosphoproteins indicated that CAV1 may play a central role in phosphorylation-mediated signaling, cell survival and apoptosis, and adhesion and migration dynamics, with additional enrichment in developmental and immune regulatory processes, collectively underscoring the potential contribution of these interaction networks to tumor progression and microenvironmental regulation (Supplementary Table S13).

## 3. Discussion

Post-translational modifications, such as phosphorylation, critically modulate protein function and signaling networks in mammalian cells. Caveolin-1 (CAV1) has emerged as a central regulator of membrane biology through its dynamic phosphorylation. Accumulating evidence highlights the significance of CAV1 phosphorylation, particularly at conserved residues, in orchestrating diverse cellular processes, including membrane trafficking, signal transduction, and oncogenic transformation [33,34,35]. CAV1 is a key structural protein of caveolae, which are plasma membrane invaginations that regulate signaling, endocytosis, and lipid transport. It modulates numerous cellular processes and is implicated in various diseases through pathways such as Mitogen-Activated Protein Kinase (MAPK), Nuclear Factor Kappa-light-chain-enhancer of Activated B Cells (NF-κB), Transforming Growth Factor Beta (TGF-β/Smad), and eNOS/nitric oxide (NO) [21]. Despite substantial advances in characterizing CAV1 as a caveolae scaffolding protein, the site-specific regulatory landscape of its phosphorylation remains incompletely defined, partly due to the complexity and heterogeneity of the large-scale datasets required for systematic study of phosphorylation dynamics. The present study addresses this gap by integrating curated phosphoproteomic datasets through a co-regulation analysis framework, enabling the identification of predominant CAV1 phosphosites and their associated signaling networks. We performed a phosphoproteomic analysis of curated datasets for CAV1, identifying CAV1_Y14 and CAV1_S37 as the most frequently perturbed phosphosites in the differential datasets. Phosphosite co-occurrence within proteins is widely recognized as an indicator of coordinated functional regulation, often reflecting shared upstream kinases or involvement in common signaling pathways [36,37]. The strong positive co-regulation observed between S37 and S9 suggests that these residues may function as a coordinated phosphorylation module, collectively contributing to CAV1-mediated cellular processes. Such closely coupled phosphosites typically reflect synchronized regulatory mechanisms rather than independent modification events, as co-phosphorylation patterns have been shown to delineate functional signaling relationships and protein interaction networks. Moderate associations among other sites, including Y6, Y25, and Y14, likely represent context-dependent or stimulus-specific signaling relationships, consistent with evidence that phosphosite co-regulation varies across biological conditions and signaling contexts [31,38]. This integrated co-regulation analysis provides insights into the complex phosphorylation dynamics of CAV1, highlighting distinct layers of regulation that may underpin its multifaceted roles in cellular signaling and function.

Our analysis identified the CAV1 predominant phosphosites, Y14 and S37, as central modulators of caveolar assembly and signaling networks through their interactions with key regulatory proteins. Specifically, CAV1_Y14 and CAV1_S37 associate with CAVIN1 (PTRF), facilitating lipid-dependent caveolae formation by enabling CAV1 recruitment and CAVIN1-mediated stabilization of caveolar domains. Disruption of this axis correlates with aberrant CAV1 localization, increased metastatic potential, and drug resistance [39,40,41,42,43]. Phosphorylation of MET at Y1234 and Y1003, particularly MET_Y1234, activates Phosphoinositide 3-Kinase–Protein Kinase B (PI3K–AKT) oncogenic signaling and is closely linked to CAV1_Y14 phosphorylation within caveolae, suggesting that CAV1_Y14 integrates MET-driven pathways with cytoskeletal remodeling and focal adhesion dynamics in breast cancer subtypes [44,45,46]. Additionally, EPHA2_Y772 co-regulates CAV1_Y14, where CAV1 scaffolding localizes EPHA2 to caveolae, sustaining ligand-independent AKT signaling that promotes angiogenesis and tumor cell migration; perturbation of this interaction attenuates tumor progression [47,48,49,50,51]. The association of CTNNB1_Y489 with CAV1_Y14 highlights a regulatory axis in which CAV1 stabilizes β-catenin at adherens junctions under physiological conditions and scaffolds Wnt/β-catenin signaling in pathological contexts, facilitating epithelial–mesenchymal transition, metastasis, and chemoresistance [52,53,54,55]. CAV1_Y14 also interacts with ERBB2_Y877, regulating HER2 membrane availability via caveolae-mediated trafficking and potentiating HER2-driven signaling, which influences tumor progression and therapeutic response [53,56,57,58]. The bidirectional regulation between CAV1_Y14 and LYN_Y32 modulates receptor organization and immune signaling, with dysregulation contributing to fibrosis and cancer [59,60,61]. Furthermore, the association of CAV1_Y14 with Dystroglycan 1 (DAG1) and Flotillin 1 (FLOT1) supports caveolar stability and AKT signaling, promoting tumor progression [62,63,64,65,66,67,68,69]. The coupling of CAV1_Y14 with Synaptosome Associated Protein 23 (SNAP23_S110) links vesicular trafficking to caveolae biogenesis, coordinating membrane fusion and transport processes [70,71,72]. In contrast, CAV1_S37 co-regulates FYN_S21, forming a caveolar complex independent of Y14 phosphorylation that controls barrier integrity and stress signaling [73,74,75,76], and associates with Solute Carrier Family 1 Member 5 (SLC1A5_S493) to facilitate glutamine transport and mTORC1 signaling, thereby driving tumor growth [77,78,79,80].

Phosphorylation at CAV1_Y14 functions as a pivotal molecular switch orchestrating diverse context-dependent cellular responses, including apoptosis, autophagy, tumor progression, and endothelial permeability, by modulating mitochondrial signaling, metabolic reprogramming, and membrane dynamics [81,82,83,84,85,86,87]. The Y14-centered network is enriched in pathways governing signal transduction, cytoskeletal remodeling, cell adhesion, and oncogenic receptor signaling, underscoring its role in coordinating caveolae-dependent communication and dynamic cellular adaptation to stress. Conversely, the S37-associated network, although less characterized, exhibits a distinct functional profile enriched for the regulation of nucleic acid metabolism, RNA processing, and negative control of biosynthetic pathways, suggesting that S37 phosphorylation modulates downstream transcriptional and metabolic responses rather than initiating signaling cascades. This functional segregation supports a model in which CAV1 phosphosites define discrete regulatory layers: Y14 mediates membrane-proximal signal propagation and cytoskeletal dynamics, whereas S37 acts as a modulatory node to refine cellular outputs. Collectively, these findings highlight the central role of site-specific CAV1 phosphorylation in integrating oncogenic signaling, membrane organization, and cellular adaptation, providing a refined framework for understanding CAV1’s multifaceted functions in cancer biology and membrane dynamics. Targeted experimental validation through time-resolved phosphoproteomics, site-directed mutagenesis, kinase inhibitor perturbation studies, and co-immunoprecipitation assays is therefore essential to confirm the mechanistic underpinnings and potential therapeutic relevance of these phosphorylation-dependent regulatory networks.

## 4. Materials and Methods

### 4.1. Assembly of the Global Phosphoproteomics Datasets with Class-1 Phosphosites in CAV1

The PubMed results for the MeSH terms “phosphoproteomics” OR “phosphoproteome” NOT “Plant” NOT “Review” were screened for published human cellular level global phosphoproteome datasets to identify Class-I phosphosites (localization probability ≥ 75% or A-score > 13) of CAV1. A fold change threshold of ≥1.3 for upregulation and ≤0.76 for downregulation, along with a *p*-value < 0.05, was used to define differential expression of Class-I phosphosites. The screened datasets were classified into Profiling (quantitative datasets comparing experimental conditions to controls) and Differential (qualitative datasets in biological or experimental conditions that list the identified phosphosites). These datasets were categorized using the phosphosite enrichment method (STY/ST/Y). To ensure uniformity, all proteins were mapped to their corresponding HUGO Gene Nomenclature Committee (HGNC) gene symbols [88] (downloaded on 30 May 2023), and their individual phosphosites in each dataset were mapped to their corresponding UniProt accession numbers using an in-house mapping tool [89]. For ease of understanding, terms used in the analysis are defined as follows: datasets—the individual biological/experimental conditions in a study; profile datasets—test conditions and control as individual datasets; quantitative datasets—biological/experimental conditions (test) versus their corresponding control; frequency—the number of datasets where CAV1 phosphosites are identified; predominant phosphosites—most repeated phosphosite which is identified across different experimental conditions.

Due to limited and inconsistent availability of raw mass spectrometry data across studies, analyses were performed using processed phosphoproteomic result tables obtained from Supplementary Materials of published studies and, where available, public repositories. These tables include identified phosphosites along with their corresponding quantitative or qualitative annotations. Therefore, data integration was conducted at the level of reported phosphosite identification and differential regulation (upregulated/downregulated), as defined within each individual study. Raw mass spectrometry files were not reprocessed.

To address the heterogeneity arising from differences in phosphopeptide enrichment strategies, instrumentation, and data processing pipelines across studies, we applied uniform inclusion and filtering criteria, including restriction to Class-1 phosphosites, standardization of protein identifiers (HGNC gene symbols), harmonization of phosphosite annotations (UniProt accession numbers), and implementation of frequency-based thresholds with replication across independent studies.

### 4.2. Identification of the Predominant Phosphosites in CAV1

Class-1 phosphosites of CAV1 were extracted from the cellular phosphoproteome datasets. The number of individual datasets in which these phosphosites were detected in qualitative profile datasets and identified as differentially expressed in quantitative differential datasets, considering their frequency, was ranked. Phosphosites that were consistently detected in both qualitative and quantitative datasets were classified as predominant phosphosites of CAV1. Notably, phosphosites that were not frequently detected or not reported as Class-1 sites within the phosphoproteome datasets were excluded from the analysis [90]. Each CAV1 phosphosite detected in the qualitative profiles and quantitative differential datasets was ranked according to its subsequent frequency of detection in different experiment conditions. Among them, the phosphosites with the highest frequency were selected as the predominant phosphosites representing CAV1 in global phosphoproteomics. A lollipop plot was generated to visualize the frequency of phosphosites in CAV1 within the assembled qualitative and quantitative data using an R/Bioconductor package, trackViewer (v1.47.0).

### 4.3. Analysis of Co-Occurrence of Phosphorylations Within CAV1

The co-occurrence of each phosphosite representing CAV1 is observed to determine the co-regulated patterns between the identified CAV1 phosphosites. The changes in the phosphorylation patterns were categorized as up-regulated (U) or down-regulated (D), and the frequencies of the four possible co-regulation patterns, U(C1)U(C2), U(C1)D(C2), D(C1)D(C2), and D(C1)U(C2) were independently quantified. The (C1) and (C2) represent the two different phosphorylation sites of CAV1. To analyse the co regulation patterns, we applied the ratio [∑(nU(C1)U(C2) + nD(C1)D(C2))/∑(nU(C1)D(C2) + nD(C1)U(C2))] to evaluate their positive co-regulation and [∑(nU(C1)D(C2) + nD(C1) U(C2))/∑(nU(C1)U(C2) + nD(C1)D(C2))] to evaluate their negative co-regulation. Higher ratio values were interpreted as indicating a strong co-regulation between CAV1 phosphosite pairs. The degree of dependency between each phosphosite was determined using this co-occurrence plot to observe how closely they were interlinked.

### 4.4. Analysis of Differential Expression of Phosphosites in Other Proteins (PsOPs) Co-Regulated with Predominant Phosphosites of CAV1

Phosphosites in other proteins exhibiting positive or negative co-regulation with predominant CAV1 phosphosites were classified according to their co-regulation patterns across the differential datasets. CAV1 phosphorylation sites were then systematically paired with phosphosites from other proteins to delineate their differential co-regulation patterns across studies. Each co-regulation pattern was denoted using paired category codes (UU, DD, UD, and DU) to describe the regulatory behavior of the two components. In each code, the first position represents the regulation status of CAV1, while the second corresponds to the regulation status of phosphosites from other proteins (PsOPs) that were differentially co-regulated with CAV1, with “U” indicating up-regulation and “D” indicating downregulation. Phosphosites from other proteins that were positively co-differentially regulated with CAV1 were classified as UUDD, whereas those that were negatively co-differentially regulated were categorized as UDDU [89,91,92].

### 4.5. Filtering of High-Confidence Phosphosites Associated with Co-Phosphoregulated Proteins of CAV1

To assess the likelihood and confidence of co-regulation within the total quantitative differential datasets, we calculated the number of datasets and/or conditions in which either the predominant phosphosite or the phosphosites in other proteins were co-detected as differentially regulated, as detailed in previous studies [91,92]. The statistical significance of this co-regulation was assessed using a one-sided FET performed on a contingency table to illustrate the association between CAV1 and its respective protein phosphosites.

Fisher’s Exact Test (FET):

$$\sum p=\frac{\left(a+b\right)!\left(c+d\right)!\left(a+c\right)!\left(b+d\right)!}{n!}\sum_{i}\frac{1}{a_{i}!b_{i}!c_{i}!d_{i}!}$$

In the FET contingency table, letters denote the following counts:

a—Number of experimental conditions in which neither phosphosite within a pair was detected.

b—Number of conditions in which only one of the two phosphosites was detected, irrespective of whether it was upregulated or downregulated.

c—Number of conditions demonstrating negative co-regulation between paired phosphosites (i.e., one site upregulated while the other is downregulated).

d—Number of conditions demonstrating positive co-regulation between paired phosphosites (i.e., both sites exhibiting either upregulation or downregulation simultaneously).

Phosphosite pairs exhibiting an FET *p*-value below 0.05 in either the positively co-regulated (UUDD) or negatively co-regulated (UDDU) categories were selected for further analysis, indicating significant co-regulation patterns. A ≥10% frequency ratio cutoff was applied to retain only phosphosites demonstrating consistent co-regulation across multiple datasets. To further minimize the potential biases from repeated experimental conditions or frequently recurring treatments, experimental code and PMIDs count thresholds (≥3) were applied, which means detection in at least three independent studies and three unique experimental conditions. Together, these criteria define high-confidence, reproducible co-regulation patterns.

### 4.6. Extraction of Upstream Kinases and Protein–Protein Interaction Partners of CAV1

The experimentally validated upstream kinases, along with their phosphosites, associated with CAV1 were curated from databases such as PhosphoSitePlus [93] (downloaded on 22 May 2023), Phospho.ELM 9.0 [94] (downloaded on 24 May 2023), and RegPhos 2.0 [31] (downloaded on 24 May 2023). Upstream kinases specific to CAV1 were predicted using multiple resources, such as NetworKIN [95] (predicted on 4 January 2023), Automatic Kinase-specific Interactions Detection (AKID) [96] (predicted on 24 May 2023), and the in vitro Kinase-to-Phosphosite database (iKiP-DB) (HTP) [97], and were assembled for kinase-substrate relationship analyses. API support was used to process the entire RefSeq protein sequences because most prediction tools require individual protein sequences as inputs. Additionally, all kinases of CAV1 phosphosites derived from synthetic peptide screening by Johnson et al. (2023) [27] were incorporated (90th percentile cutoff). The protein–protein interactors of CAV1 were extracted from several databases, such as HPRD [98], BIND [32], BioGRID [30], ConsensusPathDB version 35 [29] (downloaded on 22 May 2023), CORUM [28] (downloaded on 3 March 2023), and RegPhos 2.0 [31] (downloaded on 24 May 2023).

### 4.7. Data Visualisation

Lollipop plots illustrating the phosphorylation patterns of CAV1 phosphosites were generated using the R/Bioconductor package trackViewer (v1.47.0) [99]. The phosphosite distribution across the qualitative profile datasets was visualized using the Python (v3.13.0) packages Matplotlib (v3.10.0) and Pandas (v2.3.3). RAWGraph 2.0 [100] was used to plot circular dendrograms depicting the correlations between the interactors. Additional visualization and figure editing were performed using Adobe Illustrator (2020) (v24.3.0.569).

## 5. Limitations

The integration of large-scale phosphoproteomic datasets in this study provides a comprehensive framework for identifying biologically relevant phosphorylation events, including those of low individual frequency, across diverse experimental systems. However, several methodological and interpretive limitations must be acknowledged. As this analysis relies on processed phosphoproteomic result tables from diverse published studies rather than on the standardized reprocessing of raw mass spectrometry files, inherent variability in dataset quality, enrichment efficiency, and quantitative annotation cannot be fully normalized. Although stringent inclusion criteria—including restriction to Class I phosphosites (localization probability ≥ 75% or A-score ≥ 13), frequency-based thresholds, and cross-study replication requirements—were applied to mitigate this variability, residual heterogeneity arising from differences in enrichment platforms, instruments, and informatics pipelines cannot be entirely excluded. The phosphosite co-regulation analysis in MS-based phosphoproteomics is susceptible to technical confounding factors, including stochastic peptide sampling, variable ionization efficiency, and enrichment biases. To minimize the contribution of single-study technical artefacts, our analysis emphasizes recurrent co-differential regulation patterns across multiple independent datasets rather than single-dataset observations.

Furthermore, protein abundance may act as a confounding factor in co-regulation analysis, as coordinated changes in parent protein levels may drive apparent co-regulation of phosphosites within and across proteins independently of site-specific phosphorylation dynamics. Due to the lack of uniformly available protein-level quantification data across the assembled datasets, this confound could not be systematically corrected for in the present analysis. All co-regulation patterns should therefore be interpreted with this limitation in mind, and findings should be validated in systems where both phosphosite-level and protein-level data are available. All co-regulatory relationships identified in this study are correlational in nature. Co-regulation does not establish causality, directionality, or direct mechanistic interaction between the identified phosphosites or proteins. The functional significance of many co-regulated phosphosites, particularly for the less-characterized CAV1_S37 site, remains to be established through direct biochemical validation. Finally, potential biases arising from dataset heterogeneity and variable experimental platforms, while mitigated through rigorous multi-criteria filtering and cross-study replication requirements, cannot be entirely excluded. Targeted experimental approaches—including time-resolved phosphoproteomics, site-directed mutagenesis, kinase inhibitor perturbation studies, and co-immunoprecipitation assays—are essential to validate the identified co-regulation networks and elucidate the mechanistic basis of site-specific CAV1 phosphorylation in health and disease.

## Data Availability

The original contributions presented in this study are detailed in the article and the Supplementary Materials. Further inquiries may be directed to the corresponding author.

## Supplementary Materials

The following supporting information can be downloaded at: https://www.mdpi.com/article/10.3390/ijms27104326/s1.
